# Mortality Associated with Night and Weekend Admissions to ICU with On-Site Intensivist Coverage: Results of a Nine-Year Cohort Study (2006-2014)

**DOI:** 10.1371/journal.pone.0168548

**Published:** 2016-12-29

**Authors:** Vincent Brunot, Liliane Landreau, Philippe Corne, Laura Platon, Noémie Besnard, Aurèle Buzançais, Delphine Daubin, Jean Emmanuel Serre, Nicolas Molinari, Kada Klouche

**Affiliations:** 1 Department of Intensive Care Medicine, Lapeyronie University Hospital, Montpellier, France; 2 School of medicine, Montpellier University, Montpellier, France; 3 Department of Nephrology-Transplantation, Lapeyronie University Hospital, Montpellier, France; 4 Department of Statistics, Lapeyronie University Hospital, Montpellier, France; 5 PhyMedExp, Centre National de la Recherche Scientifique (CNRS 9214) - Institut National de la Santé et de la Recherche Médicale (INSERM U-1046), Montpellier University, Montpellier, France; University of Colorado Denver, UNITED STATES

## Abstract

**Background:**

The association between mortality and time of admission to ICU has been extensively studied but remains controversial. We revaluate the impact of time of admission on ICU mortality by retrospectively investigating a recent (2006–2014) and large ICU cohort with on-site intensivist coverage.

**Patients and Methods:**

All adults (≥ 18 years) admitted to a tertiary care medical ICU were included in the study. Patients' characteristics, medical management, and mortality were prospectively collected. Patients were classified according to their admission time: week working days on- and off-hours, and weekends. ICU mortality was the primary outcome and adjusted Hazard-ratios (HR) of death were analysed by multivariate Cox model.

**Results:**

2,428 patients were included: age 62±18 years; male: 1,515 (62%); and median SAPSII score: 38 (27–52). Overall ICU mortality rate was 13.7%. Admissions to ICU occurred during open-hours in 680 cases (28%), during night-time working days in 1,099 cases (45%) and during weekends in 649 cases (27%). Baseline characteristics of patients were similar between groups except that patients admitted during the second part of night (00:00 to 07:59) have a significantly higher SAPS II score than others. ICU mortality was comparable between patients admitted during different time periods but was significantly higher for those admitted during the second part of the night. Multivariate analysis showed however that admission during weeknights and weekends was not associated with an increased ICU mortality as compared with open-hours admissions.

**Conclusion:**

Time of admission, especially weeknight and weekend (off-hour admissions), did not influence the prognosis of ICU patients. The higher illness severity of patients admitted during the second part of the night (00:00–07:59) may explain the observed increased mortality.

## Introduction

In critical care settings, optimal treatment availability should be ensured for all patients on a 24-hour/7 days basis. Outcome of such patients depends mainly on their initial treatment that involves care teams mostly on unscheduled times of admissions [1–4]. Indeed, critically ills are often admitted to ICU during nights, weekends, holidays, off hours [5] when the intensity of care and medical staff is significantly reduced. During these time periods, physical and cognitive abilities of medical and paramedical staff may be, in addition, impaired due to sleep deprivation especially during night-time [6]. Moreover, critically ill patients usually have organ failures requiring the implementation of complex diagnostic and therapeutic procedures. These measures are urgent in most cases and cannot be postponed to on-hours. During off-hours, the lack of an early detection of patients at risk may also induce a longer delay to their admission to ICU. This disparity in patient care over time would induce a significant impact on ICU patients’ prognosis [7].

Several studies have investigated the influence of admission time on patients’ outcome. A significantly worse outcome was observed in many acute diseases including myocardial infarction and stroke when hospital admission occurred at night or over the weekend [8–12]. Then, it is a common belief that patients admitted to ICU would have a higher risk of death during off-hours. However, data related to critically ill patients remain contradictory [13–19]. While some studies demonstrated a significant association between ICU mortality and off-hours [14], others found an association only with nightshift [15, 16] and others did not find any impact of admission time on ICU mortality [19]. Discrepancy between these reports relates to differences in organization of work shifts, intensivist coverage on site, ratio of caregivers to patient, different definitions of open hours, closed or ICU “without walls”… The organisational care in ICUs has changed in the last recent years with improvement in both medical and paramedical staffing at least in western countries. In our ICU, according to French Law, medical staff has been upgraded with obligatory rest periods and a coverage intensivist on site. Since 2006 we have prospectively collected data of all patients admitted to our unit including admission time, severity score and ICU mortality. We therefore carried this study in order to reassess the potential effect of admission time on ICU mortality. We recruited our patients over 9 years from 2006 to 2014 with stringent medical organization and definitions of time of admission of patients.

## Methods

### Setting and organisation

This prospective observational cohort study was carried out in a medical ICU of an academic tertiary care hospital in Montpellier, France. This 12-bed medical ICU admitted an average of 270 patients per year. Critical care unit team included 6 attending intensivists, 4 residents (critical care or other speciality fellows), medical students, nurses, and respiratory therapists. Intensivists and residents staffed the ICU 24h per day and every day of the week. During open hours, 2 teams provided ICU medical coverage: each including a senior intensivist and a resident and taking care of 6 bed-patients. The nurse-to-patient ratio was maintained at 1:3 every time of any day. Imaging technical platform and surgical operating room were available on a 24-hour and 7-days basis. Admissions may occur at any time of the day and the night. This organisation was maintained all along the study period and was comparable to the other ICUs of our hospital.

We defined two periods of ICU admissions: on- and off-hours periods. On-hours or open-hours admissions included time period from Monday to Friday from 8:00 a.m. to 5:59 p.m. at the exception of holidays. During on-hours admissions, almost the entire Unit personnel members were present leading to the highest level of medical presence (at least 2 intensivists and 2 residents). Patients admitted during on-hours were considered as reference group. Off-hour admissions included night-time (6:00 p.m. to 7:59 a.m.), weekend (from Saturday 08:00 a.m. to Monday 7:59 a.m.) and holidays’ admissions. Holidays were those officially recognised by the French Republic. During off-hours, medical team was reduced and included one intensivist and one resident.

### Study population

All patients older than 18 years consecutively admitted to the ICU over 9-year period from January 2006 to December 2014 were included in the study. Patients who had to undergo a limitation of therapeutic effort (LTE) during their ICU stay were excluded from the analysis. Only the first ICU admission of each patient was included. Data were prospectively collected and reported in a computer Excel spread sheet database. They were recorded on a daily basis by the intensivist in charge of the patient. Data accuracy and exhaustiveness were checked before archiving paper folders. Data were analysed and stored in an anonymous way and are not traceable to any patient. The Institutional Review Board (Comité de protection des personnes: CPP CHU Montpellier) approved the study and waived the need for informed consent.

### Data collection

The following data were extracted for each patient: age and sex, time and date of ICU admission, reason for admission, and Body Mass Index (BMI). Severity of the disease was assessed 24 hours after admission using the simplified acute physiology score (SAPS) II [20]. The requirement for invasive mechanical ventilation, renal replacement therapy (RRT) and for vasoconstrictive agents was recorded. ICU length of stay (LOS) and ICU survival were recorded.

ICU mortality was the primary end point of the study.

### Statistical analysis

The statistical analyses were performed using the R 2.15.1 (The R Foundation for Statistical Computing, Vienna, Austria) software. We first performed a descriptive analysis by computing frequencies and percentages for categorial data; and means or medians, standard deviations, quartiles and extreme values for continuous data. We also checked for the normality of the continuous data distribution using the Shapiro-Wilk's tests. Continuous variables were compared using two-tailed Student t-test or two-tailed Mann-Whitney-Wilcoxon’s test when appropriate. Fisher exact and Chi 2 tests were used to compare categorial variables. To analyze the factors associated with the in-ICU survival, the Cox proportional hazards regression model was used in both univariate and multivariate models. ICU survival was calculated from the time of admission to the date of death from any cause or the date of ICU discharge. A specific potential association between time of admission and ICU survival was investigated. The proportional hazard assumption was tested and met for each variable of interest. Results were expressed as hazard ratios and 95% confidence intervals. Survival curves were generated using the Kaplan-Meier methodology. A value of p < 0.05 was considered as significant.

## Results

During the study period, 2,894 patients were admitted to the ICU. After the exclusion of 464 patients (16%) who underwent a LTE, and 2 patients for missing data, 2,428 patients were enrolled in the study.

**Fig 1:**
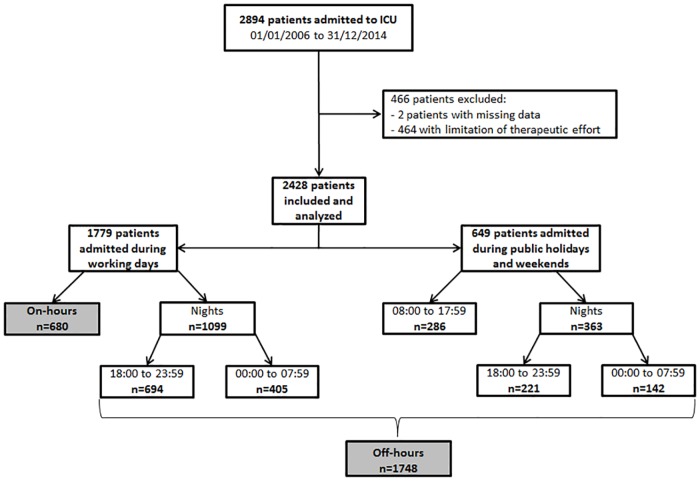
Flow-chart of the study population.

The study flow-chart is shown in Fig 1. Among the population analysed, 680 (28%) patients were admitted during on-hours and 1,748 (72%) during off-hours. Most of admissions (1,462/2,428: 60%) occurred during night-time period: 915 (38%) patients were admitted during the first part (18:00–23: 59), and 548 (22.5%) during the second part of the night (00:00–07:59). Six hundred forty-nine patients were admitted during weekends and holiday days.

Patient's characteristics, management, ICU LOS and mortality are summarized in Table 1. Population was predominantly male (62%) with a mean age of 59±18 years. Admissions were mainly medical with primarily respiratory (40.7%) and neurological (17.4%) causes. High mean SAPS-II scores underlined the severity of the patients’ illnesses. During ICU stay, a high proportion (60%) of patients was on invasive mechanical ventilation, 32.5% necessitated a catecholamine support, and 7% a RRT. The median ICU LOS was 6 days and ICU mortality rate was 14%, which is lower than the mortality predicted according to SAPSII.

**Table 1:** Demographic and epidemiological characteristics of the studied population at the admission to the ICU; management, ICU length of stay and mortality. BMI: body mass index; SAPS II: simplified acute physiology score II; ICU: intensive care unit; LOS: length of stay; “SAMU”: **S**ervice d’**A**ide **M**édicale **U**rgente: mobile emergency team.

Patient characteristics	n = 2428
**Age** (years), mean (SD)	59±18
**Male**, n (%)	1515 (62.6)
**BMI** (Kg/m^2^), median (25–75%)	24.8 (21–29)
**SAPS II score**, median (25–75%)	38 (27–52)
**Admission source**, n (%)	
Emergency department	672 (27.7)
Medical ward	650 (26.8)
Surgical ward	276 (11.4)
Transfer from other hospital	619 (25.5)
Mobile emergency team “SAMU”	202 (8.3)
Other	9 (0.3)
**Reason for ICU admission**, n (%)	
Respiratory	988 (40.7)
Cardiovascular	360 (14.8)
Neurologic	423 (17.4)
Infectious disease	216 (8.9)
Other medical	322 (13.3)
Postoperative	119 (4.9)
**Need for**, n (%)	
Invasive mechanical ventilation	1449 (59.7)
Vasopressor therapy	790 (32.5)
Renal replacement therapy	162 (6.7)
ICU LOS (days), median (25–75%)	6 (3–13)
**ICU mortality**, n (%)	332 (13.7)
**Predicted ICU mortality, %**	21.3

### Comparison of different groups according to the period of admission

The comparison between patients admitted during on-hours and off-hours is displayed in Table 2. The 2 groups were comparable in terms of demographic and epidemiologic characteristics, severity of illness and support care. Patients were more frequently admitted from the emergency department in the off-hours group (31%) than in the on-hours group (20%). Duration of mechanical ventilation and ICU LOS were significantly longer for patients admitted during on-hours than for those admitted during off-hours (7 versus 5 days, p<0.001 and 8 versus 7 days; p<0.01 respectively). ICU mortality was however comparable between patients admitted during on- and off-hours and reached approximately 14%.

**Table 2:** Comparison of patients admitted during on-hours and off-hours. BMI: body mass index; SAPS II: simplified acute physiology score II; ICU: intensive care unit; LOS: length of stay; “SAMU”: **S**ervice d’**A**ide **M**édicale **U**rgente: mobile emergency team.

	On-hours	Off-hours	*p*
**Time of ICU admission**	Monday to Friday. 08:00 to 17:59	Weeknights, weekends and public holidays	
**No. of patients**, n (%)	680 (28)	1748 (72)	
**Age**, mean (SD), years	58.6±17.48	59±18	0.58
**Male**, n (%)	429 (63)	1085 (62)	0.17
**BMI** (Kg/m^2^), median (25–75%)	24.9 (21.2–29)	24.7 (21.3–29.3)	0.89
**SAPS II score**, median (25–75%)	38 (26.25–51)	38 (27–53)	0.41
**Admission source**, n (%)			**<0.0001**
Emergency department	135 (19.8)	537 (30.7)	
Surgical ward	61 (9)	141 (8)	
Medical ward	221 (32.5)	429 (24.5)	
Mobile emergency team “SAMU”	64 (9.4)	212 (12.1)	
Transfer from other hospitals	195 (28.7)	423 (24.2)	
**Reason for ICU admission**, n (%)			0.079
Respiratory	302 (44.4)	687 (39.3)	
Cardiovascular	98 (14.4)	261 (14.9)	
Neurologic	102 (15)	321 (18.4)	
Other medical	140 (20.6)	398 (22.8)	
Postoperative	38 (5.6)	81 (4.6)	
**Need for**, n (%)			
Invasive mechanical ventilation	407 (59.8)	1041 (59.6)	0.93
Vasopressor therapy	222 (32.6)	567 (32.4)	0.96
Renal replacement therapy	53 (7.8)	108 (6.2)	0.18
**ICU LOS** (days), median (25–75%)	7 (3–15)	5(3–13)	**0.0009**
**Ventilation duration** (days), median (25–75%)	8 (3–20)	7 (3–15)	**0.0012**
**ICU mortality**, n (%)	95 (13.97)	236 (13.5)	0.84
**Predicted ICU mortality** (%)	21.3	21.3	

We compared patients admitted during working day nights and those admitted during weekends and holidays to the reference group (patients admitted on on-hours during working days). The former group did not differ from the reference group in terms of age, sex, BMI, and SAPS II scores but it presents different features. Patients admitted during nightly working days were preferentially transferred from emergencies, had significantly shorter duration of mechanical ventilation, and reduced ICU LOS than the on-hours group. Nevertheless, ICU mortality rate remained similar between the two groups, ranging from 13% to 14% (p = 0.56) (Table 3). Similarly, patients admitted during weekends and holidays did not show any differences with the reference group except a higher proportion of patients from the emergency department and a shorter duration of mechanical ventilation (6.5 versus 8 days, p = 0.018). ICU mortality was again comparable to on-hour patients group (14.5% versus 15%, p = 0.81). These results are summarized in Table 3.

**Table 3:** Comparison of patients admitted during on-hours to those admitted during weeknights, and to those admitted during weekends and public holidays. Weekend admission was defined as admission between 08:00 am Sunday and 7:59 am Monday; BMI: body mass index; SAPS II: simplified acute physiology score II; ICU: intensive care unit; LOS: length of stay; “SAMU”: Service d’Aide Médicale Urgente: mobile emergency team; p: On-hours vs Week-night; p*: On-hours vs weekends and public holidays.

	On-hours	Weeknights	*p*	*Weekends and public holidays*	P*
**Time of ICU admission**	Monday to Friday, 08:00 to 17:59	Monday to Friday, 18:00 to 07:59		Friday 18:00 to Monday 07:59 and public holidays	
**No. of patients**, n (%)	680 (28)	1099 (45.3)		649 (26.7)	
**Age**, mean (SD), years	58.6±17.5	59.3±17.4	*0*.*43*	58.43±18.63	0.86
**Male**, n (%)	429 (63)	688 (62.6)	*0*.*84*	397 (61.2)	0.47
**BMI** (Kg/m^2^), median (25–75%)	24.9 (21–29)	24.8 (21–29)	*0*.*94*	24.6 (21–29)	0.80
**SAPS II score**, median (25–75%)	38 (26–51)	37.5 (27–52)	*0*.*93*	40 (27–54)	0.097
**Admission source**, n (%)			***<0*.*0001***		***<0*.*0001***
Emergency department	135 (19.8)	342 (31.1)		195 (30.6)	
Surgical ward	61 (9)	72 (6.5)		69 (10.6)	
Medical ward	221 (32.5)	260 (23.6)		169 (26)	
“SAMU”	64 (9.4)	151 (13.7)		61 (9.4)	
Transfer from other hospitals	195 (28.7)	269 (24.5)		154 (23.7)	
**Reason for ICU admission**, n (%)			***0*.*0097***		0.25
Respiratory	302 (44.4)	419 (38.1)		268 (41.3)	
Cardiovascular	98 (14.4)	152 (13.8)		109 (16.8)	
Neurologic	102 (15)	210 (19.1)		111 (17.1)	
Other medical	140 (20.6)	258 (23.5)		140 (21.6)	
Postoperative	38 (5.6)	60 (5.5)		21 (3.2)	
**Need for**, n (%)					
Invasive mechanical ventilation	407 (59.8)	639 (58.1)	*0*.*47*	402 (61.9)	0.65
Vasopressor therapy	222 (32.6)	346 (31.5)	*0*.*61*	221 (34)	0.59
Renal replacement therapy	53 (7.8)	69 (6.3)	*0*.*22*	39 (6)	0.20
**ICU LOS** (days), median (25–75%)	7 (3–15)	5 (3–12)	***0*.*0002***	5 (3–13)	0.07
**Ventilation duration** (days), median (25–75%)	8 (3–20)	5 (3–12)	***0*.*0014***	6.5 (3–15)	**0.018**
**ICU mortality**, n (%)	95 (13.97)	142 (12.93)	*0*.*56*	94 (14.48)	0.78
**Predicted ICU mortality, %**	**21.3**	**21**		**24.7**	

We then classified the study population according to time period regardless of working day or not, considering 3 groups: the first group, considered as reference group, included patients admitted from 08:00 to 17:59 whereas the second group included patients admitted from 18:00 to 23:59 and the third group admitted from 00:00 to 7:59 (Table 4). Univariate analysis showed that patients admitted during the last part of the night were transferred preferentially from the emergency department, had a significantly higher SAPS II score, were more likely to require mechanical ventilation or/and vasopressor therapy than others. As a consequence, this group of patients has the highest mortality rate (16.5%) as compared to the open-hours group (14.5%; p = 0.01) and to the group admitted during the first part of the night (11.1%; p = 0.004).

**Table 4:** Characteristics of patients admitted per time variable regardless of type of the day (working days or not). BMI: body mass index; SAPS II: simplified acute physiology score II; ICU: intensive care unit; LOS: length of stay; “SAMU”: **S**ervice d’**A**ide **M**édicale **U**rgente: mobile emergency team; MV: mechanical ventilation. p = significance as compared to group admitted from 08:00 to 17:59.

Hours of ICU admission	08:00 to 17:59	18:00 to 23:59	00:00 to 07:59	p
**No. of patients**, n (%)	966 (39.8)	915 (37.7)	547 (22.5)	
**Age** (years), mean (SD)	58.6±17.6	59±17.5	59±18	0.69
**Male**, n (%)	610 (63.1)	554 (60.5)	350 (64)	0.34
**BMI** (Kg/m^2^), median (25–75%)	24.8 (21.2–29)	24.7 (21–29.2)	25 (21.8–29.4)	0.17
**SAPS II score**, median (25–75%)	38 (26.25–51)	36 (26–50)	41 (28–56)	**0.0053**
**Admission source**, n (%)				**<0.0001**
Emergency department	207 (21.4)	250 (27.3)	215 (39.3)	
Surgical ward	89 (9.2)	134 (14.6)	53 (9.7)	
Medical ward	303 (31.4)	242 (26.4)	104 (19)	
Transfer from other hospitals	270 (28)	223 (24.4)	125 (22.9)	
“SAMU”	91 (9.4)	62 (6.8)	49 (9)	
**Reason for ICU admission**, n (%)				**0.0096**
Respiratory	429 (44.4)	347 (37.9)	212 (38.8)	
Cardiovascular	147 (15.2)	142 (15.5)	70 (12.8)	
Neurologic	144 (14.9)	153 (16.7)	125 (22.9)	
Other medical	197 (20.4)	213 (23.3)	121 (22.1)	
Postoperative	44 (4.6)	58 (6.3)	17 (3.1)	
**Need for**, n (%)				
Invasive mechanical ventilation	584 (60.5)	515 (56.3)	349 (63.8)	**0.0144**
Vasopressor therapy	312 (32.3)	273 (29.8)	204 (37.3)	**0.0128**
Renal replacement therapy	71 (7.3)	46 (5)	42 (7.7)	0.0604
ICU LOS (days), median (25–75%)	6 (3–16)	5 (3–11)	5 (2–13)	**<0.0001**
MV (days), median (25–75%)	8 (3–19.5)	6 (3–14)	7 (2–15)	**0.0006**
ICU mortality, n (%)	140 (14.5)	102 (11.1)	90 (16.4)	**0.0107**
**Predicted ICU mortality**	21.3	18.1	26.6	

Univariate analysis showed, as expected, that age, SAPS II score and life sustaining therapy (mechanical ventilation, vasopressor therapy and renal replacement therapy) were significantly associated with ICU mortality (Table 5). Admissions occurring during the last part of the night were significantly associated with a higher mortality risk than admissions occurring during open hours (HR 1.39 [CI, 1.06–1.39, p = 0.02]) whereas those occurring during summer tended to have a lower mortality risk than those occurring in spring but without reaching significance (HR 0.77 [CI, 0.56–1.04]; p = 0.09). Multivariate analysis did confirm SAPSII, mechanical ventilation, and RRT as risk factors associated with mortality but failed to demonstrate any association between ICU mortality and time admission even for admissions occurring during the last part of the night (Table 6). Adjusted hazard-ratio of admissions from 00:00 to 7:59 was at 1.24 [CI, 0.85–1.81]; p = 0.25, as compared with admissions during open hours.

**Table 5:** Univariate Cox model of factors associated with ICU mortality. * As compared with female; Ref: reference category; reduced staff: weekend and public holydays admissions; RRT: renal replacement therapy; “SAMU”: **S**ervice d’**A**ide **M**édicale **U**rgente: mobile emergency team.

Factors	Hazard-Ratio	95% IC	p
**Age > 60 years**	1.43	[1.14–1.78]	**0.002**
Male*	1.03	[0.83–1.29]	0.77
**SAPS II ≥ 40**	5.20	[3.85–7.04]	**<0.001**
**BMI ≥ 30**	0.86	[0.64–1.15]	0.30
**Admission source**			
Medical ward	Ref.	-	-
Emergency dpt.	1.19	[0.89–1.6]	0.25
“SAMU”	1.69	[1.15–2.47]	**0.007**
Surgical ward	0.66	[0.40–1.02]	0.06
**Invasive ventilation**	4.58	[2.97–7.05]	**<0.001**
**Vasopressor therapy**	3.12	[2.63–4.43]	**<0.001**
**Renal replacement therapy**	3.51	[2.73–4.50]	**<0.001**
**Admission time**			
08:00 to 17:59	Ref.	-	**-**
18:00 to 23:59	0.86	[0.67–1.11]	0.25
00:00 to 07:59	1.39	[1.06–1.81]	**0.02**
Weekend	1.05	[0.82–1.33]	0.71
Reduced staff	1.05	[0.83–1.34]	0.67
Spring	Ref.	-	**-**
Summer	0.77	[0.56–1.04]	0.09
Autumn	0.94	[0.68–1.26]	0.62
Winter	0.84	[0.62–1.14]	0.26

**Table 6:** Multivariate Cox model of factors associated with ICU mortality. Included variables in the analysis: age, sex, BMI, SAPS II, nutritional status, admission source, hospital admission category, reason for admission, life support treatments.

Factors	Hazard-Ratio	95% IC	p
**SAPS II ≥40**	2.79	[1.73–4.49]	**<0.001**
**Reason for admission**			
Other medical	Ref.	-	-
Respiratory	1.00	[0.57–1.78]	0.99
Cardiovascular	2.68	[1.72–3.93]	**<0.001**
Neurologic	1.92	[1.27–2.90]	**0.002**
Postoperative	0.49	[0.12–2.06]	0.33
**Invasive ventilation**	16.96	[4.14–69.5]	**<0.001**
**Renal replacement therapy**	2.48	[1.72–3.56]	**<0.001**
**Admission time**			
08:00 to 17:59	Ref.	-	-
18:00 to 23:59	0.95	[0.66–1.37]	0.80
00:00 to 07:59	1.24	[0.85–1.81]	0.25

**Fig 2:**
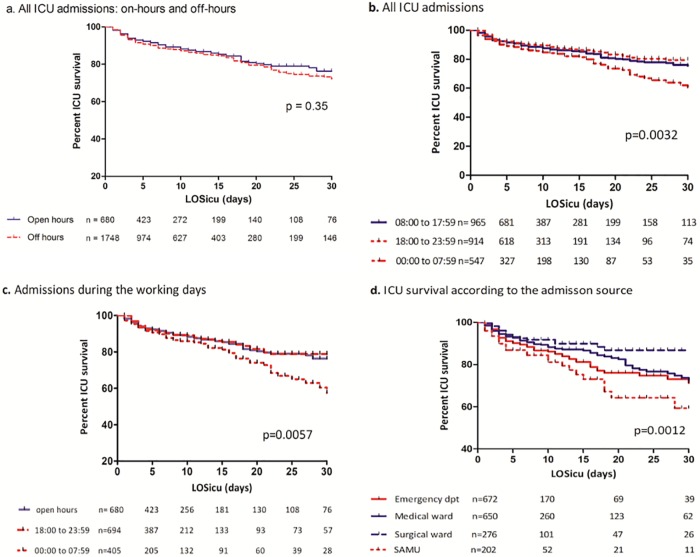
Kaplan–Meier curves of ICU survival according to the time and source of admission. Comparison of ICU survival of patients admitted during on-hours and off-hours **(a)** and of patients admitted from 08:00 to 17:59, 18:00 to 23:59, and 00:00 to 07:59 **(b)**. Comparison of ICU survival of patients admitted during weekdays according to time period of admission **(c)**. Comparison of ICU survival according to the source of admission **(d)**. LOSicu: length of stay in the ICU.

Fig 2 represents the Kaplan-Meier curves for ICU survival according to different time periods and admission source. The comparison between patients admitted during on- and off-hours showed no differences in ICU actuarial survival (Fig 2a). Analysis of all ICU admissions (Fig 2b) and of working days’ admissions (Fig 2c), showed that patients admitted during the second part of the night had a significantly higher mortality rate than others (Fig 2b and 2c). Patients transferred from the emergency department or directly by an emergency mobile team have the highest mortality rate (Fig 2d).

## Discussion

In this single-centre large cohort study, we observed that most of ICU admissions occurred during off-hours. In univariate analysis, patients admitted during the second part of the night bears the worst prognosis with a significantly higher mortality. After adjustment for confounding variables especially illness severity, night-time admission was not associated with mortality. Our observations therefore suggest that time of admission, especially weeknight and weekend (off-hour admissions), did not influence the prognosis of ICU patients.

In ICUs, diagnostic procedures, optimal treatment, and necessary staffing should be available to all patients on a 24 hour and 7 days basis. However, an increased mortality has been observed and reported during off-hours, especially during weekend [5, 21]. Several factors have been implicated in this association including reduced medical staff, higher working load, and difficult accessibility to surgical or imaging platform. Differences in patient characteristics such as disease severity have also been advocated. Though various studies have been conducted in adults [13–19,21–26] and paediatrics [27,28] to assess a link between mortality and time of patient admission, especially for those admitted during weekends, results remain however controversial. Yet, patients admitted for myocardial infarction [10], strokes [9], pulmonary embolism or aortic aneurism rupture [8], have a higher mortality rate when admitted on weekends rather than on weekdays. Also, Kuijsten et al. [14] observed a significantly higher mortality rate in adult patients admitted to ICU on weekends and night-times as compared to those admitted on weekdays. Moreover, Barnett et al. [17], in a study of 150,000 adult patients from 38 ICUs, demonstrated a higher risk-adjusted odd of in-hospital death for patients admitted on weekends as compared to those admitted on weekdays. While some studies showed the weekend impact [13, 21], others highlighted the effect of night-time admission [15,29]. The meta-analysis performed by Cavallazzi et al. [5] indicated that an increased risk of death was associated with weekends but not with night-time admissions. By analysing 1,106 ICU patients, Abella and colleagues [21] showed indeed that hospital mortality was independently associated with off-hours admission. However, in the subgroup of off-hours patients they found that ICU admission on weekends or non-working days, compared to daily night shifts, was independently associated with hospital mortality with an odd ratio at 2.30 (95%CI 1.23–4.30). In contrast, other investigators did not observe any increased risk of mortality associated with ICU admissions neither on weekends nor on nights [16,19,22] and even a better outcome for patients admitted during off-hours [23]. These contradictory results may be explained by different definitions of off-hours, organisational model in both medical and paramedical staff, different availability of diagnosis and invasive therapeutic procedures. Nurses’ staff organization, for example, is rarely reported precluding a correct appreciation [5] knowing that a correlation exists between the number of caregivers and prognosis [30–33]. Thus, our study was undertaken to evaluate the impact of on- and off-hours on mortality on a large and recent cohort taking into account to these confounding variables. An on-site senior intensivist staffed our ICU 24-hours a day and 7 days a week. Nurse to patient ratio was maintained constant over time but without considering workload and fatigue in night work. Our definition of on- and off-hours covered the presence and the number of intensivists and off-hours mainly stand for reduced medical staff. Diagnostic and therapeutic procedures, even the most complex, were available 24 hours a day. In these conditions, we found a higher mortality only for patients admitted during the second part of the night but this mortality was not associated to time period admission but rather with disease severity.

Obviously, the comparison of our results to previous studies is somewhat difficult since medical ICU organization varies from one country to another and even from one hospital to another within the same country. Ju and colleagues [15] conducted their study in a Chinese hospital in which medical staff included, during the night, only a non-specialized resident on-site while an intensivist was on the phone. Two French studies [23,34] described a medical organization close to ours: the multicenter study showed a protective, but negligible, effect of nightly admissions [23].

Whether the presence of an intensivist during off-hours or the unit organisation influences ICU mortality remains however questioned [35–40]. Indeed, it has been shown in academic high density ICUs that the presence of senior intensivists during night-time did not improve patient’s survival [38,39]. The meta-analysis carried out by Wilcox et al. [41] showed a significant improvement on ICU survival with high intensity staffing versus low intensity staffing (no intensivist on coverage) (RR 0.81; 95% CI 0.68–0.96). However, on examining the 24-h intensivist model versus intensivist coverage only during the day shift, no decrease in mortality was found (RR 0.88; 95% CI 0.7–1.1). ICU survival would rather depend on organization during open hours: quality of care and the number of physicians working during daytime may influence prognosis of off-hours patients [42,43].

It is noteworthy that a majority of our patients (71%) was not admitted during open hours. Al Arabi et al. [22] and Luyt et al. [23] reported similar observations with a proportion of off-hours admitted patients varying from 65 to 69%. In such ICUs, medical teams are used to support a work overload and mortality is not impacted. On the other hand, ICU teams, who carried out admissions mostly during open hours, observed a worse outcome of patients admitted during off-hours [14,16]. In addition, patient’s severity criteria may differ according to on- and off-hours admission [24,34]. Yet, severity and mortality have been found increased [14,15] or decreased [16,24] in patients admitted off-hours. We observed that patients admitted during the second part of the night experienced an increased mortality but also had a higher disease severity. Multivariate analysis demonstrated that this increased mortality rate depends on severity score but not on admission time. Wunsch et al. [19] have also observed, in a prospective cohort, an increased mortality among patients admitted overnight and weekends that disappeared after adjustment for illness severity. Our patients came preferentially from emergency unit during off-hours whereas they came more frequently from medical or surgical wards in open-hours, as reported by others [23,24]. Patients admitted during open-hours have a longer duration of mechanical ventilation and ICU LOS suggesting a greater severity of admissions from the ward as compared to those from emergency department [44,45]. Delay to ICU admission, which is over risky, would be longer for patients in the ward because they are often placed on hold when no bed is available [45–47]. On the other hand, early detection of potentially critical patients as done in ICUs without walls may lead to improved survival rates in patients from the ward [48, 49]. In a before-after study, Abella et al [49] demonstrated that the use of a proactive strategy, allowing intensivists to intervene outside the ICU for an early detection of patients at risk, induced a significant decrease in mortality of ICU patients admitted on week-ends and holidays.

Patient's outcome depends on the physician in charge and on its capacities that vary according to admission time. In 6 ICUs in France, it was found that age, ICU experience, and religious beliefs of intensivists were significantly associated with life-sustaining treatments [34]. Effectiveness and decision-making are probably less stringent during the night or weekends; sleep deprivation and overwork diminished the medical staff’s cognitive abilities [6,50–53]. Intensivists in French ICUs have to rest during at least 11 hours after night shift. Our intensivists followed these directives explaining, at least for a part, our results. Two large multicentre observational studies involving 49 and 143 ICUs have previously demonstrated no benefits of night-time intensivists, regardless of daytime staffing model [54,55]. Others studies showed however an improved ICU mortality with night-time physician staffing [38,56].

The ratio of the number of caregivers by period to the number of admissions should be taken into consideration. Admitting a high number of patients would reduce medical and paramedical time spent on each patient and increase workload and mortality [57,58]. Neuraz et al. [58] proposed, through a multicentre study, an evidence-based threshold of 5 patients to 2 nurses and 14 patients to one physician above which there was an increase in ICU mortality. In our study, such ratios were fulfilled. We noticed however that the most severe patients were admitted between 00:00 and 07:59, a time period when fatigue usually culminates [59]. However, it was also the period with the smallest number of admissions. Similarly, workload is slightly lower on weekends (especially at night), compared to the rest of the week. Decrease in activity could counterbalance the reduced medical staffing. An early detection of patients at risk in the hospital, like experimented in some ICUs “without walls”, may be an innovative management in ICUs and may induce a significant improvement. Last, on- and off-hours, weekends and nights were differently defined in the various published reports. Weekend spans from Thursday to Friday in eastern countries like Saudi-Arabia [22] while it spans from Saturday to Sunday in the western countries.

This study has several limitations. First, it is a monocentric study. Since our organization is characterized by a “closed” activity within a high-density medical university structure as other units in our hospital, our results should not be extrapolated to community hospital units or to ICUs without on-site intensivist coverage. However, the single-centre nature of our study offers the advantage of no inter-centre organization variability. Second, mortality after ICU discharge has not been collected herein precluding any definite conclusions about the long-term impact of time of admission. Moreover, even if time of admission did not influence short outcome, its impact on others indicators, including adverse events, patient’s well-being, mistakes, accidents and their side effects that deserve interest, may be altered. Unfortunately, such outcomes have not been collected in our work. Third, the studied population was mostly medical and our results may not be applied to surgical ICUs. Indeed, in the retrospective cohort reported by Esminger and colleagues [18], weekend ICU admissions were associated with increased hospital mortality only in the subgroup of patients admitted in the surgical ICU. Last, we excluded from our analysis patients with limitations on life support because such limitations were less commonly enacted during off-hours.

## Conclusion

Our study carried out in an ICU with an intensivist on site coverage indicates that time of admission did not influence mortality. Though admissions occur mostly during off-hours, the observed increased mortality was not related to admission during the night (00:00–07:59) but to patient illness severity after correction of confounding variables. Improved medical staffing during both on- and off-hours but also organisational ICU model including interprofessional care teams and ICU protocols may limit the potential harm effect of admissions out off-office hour's.

## Supporting Information

S1 Dataset Dataset of all patients. Dataset.
(XLSX)

## Abbreviations

BMI: Body Mass Index
CI: Confidence Interval
HR: Hazard-Ratio
ICU: Intensive Care Unit
IQ: InterQuartile
LOS: Length Of Stay
LTE: Limitation of Therapeutic Effort
RRT: renal replacement therapy
SAPS: Simplified Acute Physiology Score
SD: Standard Deviation
